# Negative Association Between Mediterranean Diet Adherence and COVID-19 Cases and Related Deaths in Spain and 23 OECD Countries: An Ecological Study

**DOI:** 10.3389/fnut.2021.591964

**Published:** 2021-03-05

**Authors:** Michael W. Greene, Alexis P. Roberts, Andrew D. Frugé

**Affiliations:** ^1^Department of Nutrition, Auburn University, Auburn, AL, United States; ^2^Boshell Diabetes and Metabolic Disease Research Program, Auburn University, Auburn, AL, United States

**Keywords:** COVID-19, mediterranean diet, coronavirus, chronic disease, healthy diet, pandemic

## Abstract

In December 2019, the severe acute respiratory syndrome coronavirus 2 (SARS-Cov2) emerged in Wuhan, China, sparking the Coronavirus disease 2019 (COVID-19) pandemic. The high prevalence of nutrition-related COVID-19 risk factors including obesity, type 2 diabetes, and hypertension, suggests that healthy dietary approaches may mitigate COVID-19 related outcomes and possibly SARS-CoV-2 infection. Based on the fundamental role of nutrition in immune function and the well-documented association between Mediterranean diet consumption and risk reduction for chronic diseases that are comorbidities in COVID-19 patients, we hypothesized that there would be a relationship between Mediterranean diet adherence and COVID-19 cases and related deaths. In this perspective, we examined the association between regional adherence to a Mediterranean diet and COVID-19 cases and deaths using an ecological study design. We observed that Mediterranean diet adherence was negatively associated with both COVID-19 cases and related deaths across 17 regions in Spain and that the relationship remained when adjusted for factors of well-being. We also observed a negative association between Mediterranean diet adherence and COVID-19 related deaths across 23 countries when adjusted for factors of well-being and physical inactivity. The anti-inflammatory properties of the Mediterranean diet - likely due to the polyphenol content of the diet - may be a biological basis to explain our findings. However, there are confounding factors unrelated to dietary factors driving COVID-19 cases and related deaths across the regions in Spain and the 23 countries examined in our analysis. Our findings will need to be confirmed and further explored in cohort studies.

## Introduction

Epidemics and pandemics have shaped the historys of humanity. Deaths from pandemics have ranged from up to half of the world's population in the Post-classical period to very few deaths in the Late Modern period (1). The Coronavirus disease 2019 (COVID-19) pandemic is just the most recent of a series of epidemics and pandemics that are arising (1) likely due to environmental pressure from the expansion of human populations (2). Increasing population density and economic development, or westernization, has not only been associated with infectious diseases, but non-communicable diseases such as obesity, cardiovascular disease, and type 2 diabetes.

Consumption of a Western diet which includes high consumption of energy dense foods, such as simple sugars and fats, contributes to the development of obesity and pathophysiological chronic inflammation (3, 4) which are risk factors for the development of chronic diseases including cardiovascular disease, type 2 diabetes, and certain forms of cancer (5). In addition, obesity is associated with immunomodulatory effects related to infectious, gastrointestinal, and respiratory diseases (6). In contrast, essential micronutrients and polyphenolic compounds which are enriched in healthy diets are anti-inflammatory and may have benefits for risk reduction of communicable and non-communicable diseases (7–9). Adherence to a traditional Mediterranean diet, a plant-based diet rich in fresh fruits and vegetables, whole grains, nuts, fish and extra virgin olive oil, can reduce the risk of cardiovascular disease, type 2 diabetes, cognitive disorders, muscle atrophy and other signs of frailty (10–12). A Mediterranean diet is also rich in fiber which modulates nutrient absorption and satiety and contributes to maintaining a healthy weight (13).

Common comorbidities in COVID-19 patients include obesity, hypertension, cardiovascular disease, type 2 diabetes, chronic obstructive pulmonary disease, chronic kidney disease, cerebrovascular disease and cancer (14–16). Increased risk of COVID-19 related death - reported in a meta-analysis of studies from China and the USA - has been observed for patients with chronic renal disease (OR, 9.4; 95% CI, 3.2–27.4), cardiovascular disease (OR, 3.8; 95% CI, 2.1–6.9), hypertension (OR, 2.5; 95% CI, 2.1–3.1), type 2 diabetes (OR, 2.0; 95% CI, 1.7–2.3) (17), and in a separate analysis obesity (OR 1.7; 95% CI, 1.1–2.8) from a cohort in the USA (18). Obesity related comorbidities that increase the severity of COVID-19 include respiratory dysfunction resulting in lower lung volume, impaired gas exchange, and increased airway resistance (19). A greater association with comorbidities and in-hospital mortality has been observed in patients in America and Europe compared to Asia (20).

Because the Mediterranean diet is a dietary approach associated with overall well-being, and Mediterranean diet consumption is associated with risk reduction for the common comorbidities observed in COVID-19 patients, we hypothesized that Mediterranean diet adherence would be negatively associated with COVID-19 cases and related deaths. The absence of dietary data in COVID-19 patients precluded a direct examination of the relationship between Mediterranean diet adherence and COVID-19 cases and related deaths. Therefore, we undertook an ecological study to examine the association between regional adherence to a Mediterranean diet and COVID-19 cases and deaths.

## Ecological Analysis

To determine whether there is relationship between Mediterranean diet adherence and COVID-19 cases and deaths we first chose to examine regional data from a country with a universal healthcare system to reduce confounding factors associated other healthcare models which may lack standardization of the COVID-19 response. We also sought to examine a country for which (1) regional differences in MD adherence have been reported, (2) regional COVID-19 cases and deaths, and (3) risk-modifying factors have been reported. Based on our criteria, we examined the relationship across 17 autonomous communities (regions) of Spain between Mediterranean Adequacy Index score (21) and COVID-19 cases and deaths as of June 9, 2020 (22). In an unadjusted linear model, we observed a significant negative association between Mediterranean Adequacy Index score and COVID-19 cases (*p* = 0.023) and COVID-19 related deaths (*p* = 0.032) (Table 1). We used a multivariable linear model to adjust for social determinants of health (factors of well-being) which can influence health outcomes (23–26). The factors of well-being (income, education, housing, environment, and life satisfaction) were assessed using the Organization for Economic Co-operation and Development (OECD) Well-Being Database in the 17 regions of Spain (27). As shown in Table 1, a stronger significant negative association between Mediterranean Adequacy Index score and COVID-19 cases (*r*^2^ = 0.663) and COVID-19 related deaths (*r*^2^ = 0.729) was observed. The well-being factors of income, education, and life satisfaction added significantly (*p* < 0.05) to the relationship between Mediterranean Adequacy Index score and both COVID-19 cases and COVID-19 related deaths.

**Table 1:** Multivariable linear regression analysis assessing Mediterranean diet adherence and COVID-19 cases and deaths between regions in Spain^‡^ adjusted for well-being factors.

	**Cases^†^**				**Deaths^†^**			
	**β**	**SE**	***p*-value**	**Adj. *R*^2^**	**β**	**SE**	**p-value**	**Adj. *R*^2^**
Univariate Model			0.023^*^	0.255			0.032^*^	0.224
MAI^a^	−0.037	0.014	0.023^*^		−0.004	0.002	0.032^*^	
Multivariate Model			0.006	0.663			0.002^*^	0.729
MAI^a^	−0.032	0.014	0.045^*^		−0.003	0.001	0.043^*^	
Income^b^	0.008	0.002	0.007^*^		0.001	2.3e-04	0.001^*^	
Education^c^	−0.003	0.001	0.046^*^		−3.6e-04	1.1e-04	0.008^*^	
Housing^d^	−0.002	0.001	0.161		−3.0e-04	1.2e-04	0.034^*^	
Environment^e^	0.001	0.002	0.504		2.0e-04	1.4e-04	0.175	
Life satisfaction^f^	−0.001	5.2e-04	0.046^*^		−1.3e-04	4.9e-05	0.022^*^	

‡ *Regions of Spain; Andalusia, Aragon, Asturias, Balearic Islands, Basque Country, Canary Islands, Cantabria, Castile and Leon, Castile-La Mancha, Catalonia, Ceuta, Extremadura, Galicia, La Roja, Madrid, Melilla, Murcia, Navarra, Valencia*.

† *Cases and deaths were normalized to the population in the regions*.

* *Significant results (P < 0.05)*.

a *MAI; Mediterranean Adherence Index Scores*.

b *Income; Household disposable income per capita (in real USD PPP)*.

c *Education; Share of labor force with at least secondary education (%)*.

d *Housing; Number of rooms per person (ratio)*.

e *Environment; Estimated average exposure to air pollution in PM2.5 (μg/m^3^), based on satellite imagery data*.

f *Life satisfaction; Average self-evaluation of life satisfaction on a scale from 0 to 10*.

To further explore the relationship between Mediterranean diet adherence and COVID-19 cases and deaths, we examined Mediterranean adherence and COVID-19 cases and deaths across 23 countries in the OECD Well-Being Database for which Mediterranean Adequacy Index score (from highest to lowest: Turkey, Greece, Italy, Japan, Chile, Spain, Portugal, Israel, Finland, Norway, Iceland, United Kingdom, Ireland, France, Denmark, Sweden, Canada, Hungary, Germany, Austria, Australia, Switzerland, and United States) (28) and COVID-19 cases and deaths per million people (from highest to lowest: United Kingdom, Spain, Italy, France, Ireland, United States, Canada, Switzerland, Portugal, Chile, Germany, Denmark, Sweden, Austria, Turkey, Finland, Hungary, Norway, Israel, Iceland, Greece, Japan, and Australia) (29). As shown in Table 2, we did not observe an association between Mediterranean Adequacy Index score and COVID-19 cases or COVID-19 related deaths in an unadjusted linear model. There was also not an association between Mediterranean Adequacy Index score and COVID-19 cases when adjusted for well-being factors in the OECD Well-Being Database. However, a significant negative association between Mediterranean Adequacy Index score and COVID-19 related deaths (*r*^2^ = 0.768, *p* = 0.004) was observed when adjusted for well-being factors (income, education, housing, environment, and life satisfaction) in the OECD Well-Being Database and physical inactivity from the World Health Organization Global Health Observatory data repository (30). The well-being factors of education, housing, environment, and life satisfaction added significantly (*p* < 0.05) to the relationship between Mediterranean Adequacy Index score and COVID-19 related deaths. A sensitivity analysis was performed by removing the Mediterranean Adequacy Index score from the model. We found that the absence of the Mediterranean Adequacy Index score significantly (*p* < 0.01) weakened the adjusted square of the coefficient of multiple correlation from 0.768 to 0.500 (data not shown).

**Table 2:** Multivariable linear regression analysis assessing Mediterranean diet adherence and COVID-19 cases and deaths between Countries^‡^ adjusted for well-being factors.

	**Cases^†^**				**Deaths^†^**			
	**β**	**SE**	***p*-Value**	**Adj. R^2^**	**β**	**SE**	***p*-Value**	**Adj. R^2^**
Univariate Model			0.738	−0.042			0.817	−0.047
MAI^a^	−0.358	1.057	0.738		−0.024	0.102	0.817	
Multivariate Model			0.121	0.343			0.004^*^	0.768
MAI^a^	−1.487	2.685	0.593		−0.597	0.176	0.010^*^	
Income^b^	8.3e-05	1.3e-04	0.526		2.6e-06	7.8e-06	0.745	
Education^c^	−0.013	0.006	0.073		−0.014	0.003	0.003^*^	
Housing^d^	0.001	0.273	0.958		0.065	0.018	0.009^*^	
Environment^e^	−0.161	0.010	0.126		−0.037	0.005	0.001^*^	
Life satisfaction^f^	2.576	1.573	0.136		0.298	0.097	0.016^*^	
Physical inactivity^g^	0.066	0.076	0.406		5.2e-03	4.8e-03	0.318	

‡ *Countries; Australia, Austria, Canada, Chile, Denmark, Finland, France, Germany, Greece, Hungary, Iceland, Ireland, Israel, Italy, Japan, Norway, Portugal, Spain, Sweden, Switzerland, Turkey, United Kingdom, United States*.

† *Cases and deaths were normalized to the population in the regions*.

* *Significant results (P < 0.05)*.

a *MAI, Mediterranean Adherence Index Scores*.

b *Income; Household disposable income per capita (in real USD PPP)*.

c *Education; Share of labor force with at least secondary education (%)*.

d *Housing; Number of rooms per person (ratio)*.

e *Environment; Estimated average exposure to air pollution in PM2.5 (μg/m^3^), based on satellite imagery data*.

f *Life satisfaction; Average self-evaluation of life satisfaction on a scale from 0 to 10*.

g *Physical inactivity: Prevalence of insufficient physical activity among adults aged 18+ years (age-standardized estimate)*.

In a sub-analysis of only European countries, we observed a similar significant negative association between Mediterranean Adequacy Index score and COVID-19 related deaths (*r*^2^ = 0.771, *p* = 0.030) when adjusted for well-being factors and physical inactivity (data not shown). We next examined whether the association between Mediterranean Adequacy Index score and COVID-19 related deaths was modified by adding country wide obesity prevalence (31) to the model adjusted for well-being factors and physical inactivity. We still observed a significant negative association (*r*^2^ = 0.751, *p* = 0.012) between Mediterranean Adequacy Index score and COVID-19 related deaths; however, obesity prevalence did not significantly add to the model (data not shown).

## Discussion

In the current ecological study, we observed that Mediterranean diet adherence was negatively associated with both COVID-19 cases and related deaths across 17 regions in Spain. The relationship between Mediterranean diet adherence and COVID-19 cases and related deaths remained when adjusted for factors of well-being. We then examined the relationship between Mediterranean diet adherence and COVID-19 cases and related deaths across 23 countries for which data was available for Mediterranean diet adherence, COVID-19 cases and deaths, and factors of well-being. In the models adjusted for factors of well-being and physical inactivity we observed a negative association between Mediterranean diet adherence and COVID-19 related deaths. Our findings that the socioeconomic factors of income and education added significantly to our models is consistent with prior work that income and education are significant factors in viral infection related hospitalization (32–35) and Mediterranean diet adherence (23, 36–40). The finding that life satisfaction added significantly to the adjusted models is consistent with epidemiological evidence linking life satisfaction to risk of chronic disease and mortality (41–43). It is important to note that our findings with COVID-19 cases and deaths pre-dated use and approval of medical treatments known to reduce mortality such as dexamethasone and remdesivir (44).

In the analysis of the 23 OECD countries we observed that the United States and the United Kingdom had the 1st and 11th lowest Mediterranean diet adherence, respectively, and the 6th and 1st highest COVID-19 related deaths per million, respectively. In contrast, Italy and Spain were outliers in the association between Mediterranean diet adherence and COVID-19 related deaths in the 25 OECD countries. Italy and Spain had the 3rd and 6th highest in Mediterranean Adequacy Index score, respectively, yet high COVID-19 mortality (3rd and 2nd highest in deaths per million people, respectively). Italy and Spain along with China, Iran, Germany, France, South Korea, and Japan were the eight earliest majorly effected countries in the COVID-19 pandemic. The doubling time of COVID-19 cases in Italy during the first and second 3-week interval was greatest among the eight countries while the growth rate of COVID-19 cases from the first to second 3-week interval in Spain was greatest among the eight countries (45). Whether poor early containment was a factor in the high COVID-19 death rate is not known. Factors ranging from socio-cultural to health systems likely have contributed to the high COVID-19 death rate in Italy and Spain (46–48). Interestingly, Lombardy, Italy, a region in northern Italy which has low Mediterranean diet adherence compared to southern Italy (38, 49, 50), was the epicenter of COVID-19 cases in Europe.

The current study design does not allow for causative conclusions to be reached on our findings. However, there is a biological basis for our findings. Severe complications of COVID-19 include acute respiratory distress syndrome and death. In the subset of patients with severe complications, a hyper inflammation (a.k.a. the cytokine storm) state is observed (51) possibility due to a dysregulated macrophage response (52). It has been proposed that comorbidities including obesity, type 2 diabetes, cardiovascular disease, and hypertension contribute to the inflammation response leading to severe complications of COVID-19 (53).

Adherence to plant-based dietary approaches, including the Mediterranean diet, are associated with a reduction in the risk of developing major chronic diseases including the COVID-19 comorbidities of obesity, type 2 diabetes, cardiovascular disease, and hypertension (54–59). Low-grade chronic inflammation underlies the COVID-19 comorbidities (60). Importantly, consumption of a Mediterranean diet is associated with a reduction in the inflammation, coagulation, and endothelial dysfunction markers including c-reactive protein (CRP), interleukin 6 (IL-6), fibrinogen, homocysteine, and E-selectin (61–63) which is consistent with findings of a reduction in inflammation markers across plant-based diet consumption (64). An assessment of overall inflammatory potential of a diet, the dietary inflammatory index (DII) (65, 66) has been shown to link dietary inflammation to communicable disease and inflammation-related disease. A high DII has been reported to be associated with higher risk for stomach and intestinal illness (67) and with increased systemic inflammation and lower lung function (68). Importantly, a high DII is associated with low grade inflammation and low Mediterranean diet score (69). It has been proposed that the anti-inflammatory properties of the Mediterranean diet are due to the high polyphenol content (70–72) from fruits, vegetables and extra virgin olive oil (73). In the SUN cohort, participants with the highest Mediterranean diet adherence and polyphenol intake of flavonoids had a significantly lower incidence of cardiovascular disease events compared to participants with the lowest Mediterranean diet adherence and polyphenol intake of flavonoids (74). Consistent with these findings, it was observed in high cardiovascular disease risk patients in the PREDIMED study that the greatest reduction in plasma levels of inflammatory markers occurred in participants with the highest urinary total polyphenols (75). It should be noted that the PREDIMED study did not have full randomization to the Mediterranean diet treatment groups (76).

There are limitations to the current study. Importantly, the ecological design of the current study does not allow for direct examination of the relationship between Mediterranean diet adherence and COVID-19 cases and related deaths. The observational methodology used may be a source of errors that could lead to wrong conclusions on the association between Mediterranean diet adherence and COVID-19 cases and related deaths. Thus, causation cannot be inferred from these findings. Additionally, the possible age effect of each group (the habits and behaviors of each stage of life are different) and the period effect (events that occur over time that affect all age groups and can alter the association) are limitations. Further, the Mediterranean diet adherence data may not reflect current dietary patterns in the regions of Spain and the OECD countries examined. Even though Mediterranean diet adherence has declined in Spain and other OECD countries in the Mediterranean Sea basin compared to historical data from the 1960s, it has stabilized in the past decades (21, 28). In addition, Mediterranean diet adherence assessed at two time points in the past two decades highly correlates across countries (*r*^2^ = 0.969, *p* < 0.001) (data not shown). We acknowledge that there are confounding factors related to societal norms, cultural factors, and the governmental response to the COVID-19 pandemic and differences in health care systems across countries that may influence COVID-19 cases and deaths in Spain and the countries examined in the current study. As the COVID-19 pandemic continues our findings represent a snapshot in time of the pandemic.

Even though our findings suggest a negative relationship between Mediterranean diet adherence and COVID-19 cases and related deaths, further studies are required to examine whether Mediterranean diet consumption reduces the risk of COVID-19 and/or chronic disease risk reduction associated with Mediterranean diet consumption reduces the risk of COVID-19 death. In conclusion, the Mediterranean diet and other dietary approaches that reduce inflammation and risk for chronic disease might reduce the risk for severe COVID-19 pathology and mortality.

## Data Availability Statement

The datasets generated for this study can be obtained from the corresponding author (mwgreene@auburn.edu).

## Author Contributions

MG conceived of the work, performed data and statistical analysis, contributed to the writing, and offered critical comments. AR performed data analysis, wrote the first draft of the manuscript, and provided critical revisions to the content. AF provided critical revisions to the content. All authors contributed to the article and approved the submitted version.

## Conflict of Interest

The authors declare that the research was conducted in the absence of any commercial or financial relationships that could be construed as a potential conflict of interest.
